# Microwave-Assisted and Conventional Extractions of Volatile Compounds from *Rosa x damascena* Mill. Fresh Petals for Cosmetic Applications

**DOI:** 10.3390/molecules27123963

**Published:** 2022-06-20

**Authors:** Carla Villa, Francesco Saverio Robustelli Della Cuna, Eleonora Russo, Mohammed Farhad Ibrahim, Elena Grignani, Stefania Preda

**Affiliations:** 1 Department of Pharmacy, University of Genova, Viale Benedetto XV, 3, 16132 Genova, Italy; villa@difar.unige.it; 2 Department of Drug Sciences, University of Pavia, Via Taramelli 12, 27100 Pavia, Italy; gardy1988@gmail.com (M.F.I.); stefania.preda@unipv.it (S.P.); 3 Environmental Research Center, ICS Maugeri SPA SB, Institute of Pavia, IRCCS, Via Maugeri 2, 27100 Pavia, Italy; elena.grignani@icsmaugeri.it

**Keywords:** *Rosa x damascena* Mill., steam distillation, hydrodistillation, microwave hydrodiffusion and gravity, solvent-free microwave extraction, principal component analysis (PCA)

## Abstract

*Rosa x damascena* Mill. essential oil is mainly used in the cosmetics and perfumery industry, but it also finds application in the food industry as a flavoring agent. The chemical composition of essential oils is affected by environment, soil, harvesting technique, storage condition, and extraction methods. Nowadays, the study and design of greener, more efficient, and sustainable extractive procedures is the main and strategic focus in the chemical research and development of botanical derivatives, especially as regards fragrances and essential oils. Several technologies are available, and the best method to use depends on the desired chemicals, but conventional extractive processes are often laborious and time-consuming, involve large amounts of solvents, and may cause the partial loss of volatiles, affecting the quality of the final product. In the last decade, microwave irradiation has been successfully applied to classical techniques, often improving the general extractive efficiency and extract quality. In the present paper, as a preliminary analytical screening approach, two microwave-mediated techniques, Solvent-Free Microwave Extraction (SFME) and Microwave Hydrodiffusion and Gravity (MHG), and two conventional procedures, Hydrodistillation (HD) and Steam Distillation (SD), were applied and compared for the extraction of volatile compounds from *R. x damascena* fresh petals to highlight differences and advantages of the selected procedure and of the obtained extracts useful in a cosmetic context as fragrances or active ingredients. The chemical composition of the extracts was investigated by GC-MS and GC-FID. Sixty-one components, distributed in the four techniques, were identified. SD and HD are dominated by oxygenated terpenes (59.01% and 50.06%, respectively), while MHG and SFME extracts are dominated by alcohols (61.67% and 46.81%, respectively). A relevant variability in the composition of the extracts relating to the extraction techniques used was observed. To point out the correlation between the process and composition of the obtained natural products, principal component analysis (PCA) of the data extracted from GC-FID was used. Taking into account a cosmetic application, SFME shows several advantages when compared with the other procedures. The extract (obtained in a significantly higher amount) contains a meaningful lower level of potential fragrance allergenic compounds and quite a double amount of benzyl alcohol and 2-phenyl ethanol that can also enhance the preservative action in personal care products.

## 1. Introduction

*Rosa x damascena* Mill. (*Rosaceae*) is an ornamental rose hybrid (*Rosa gallica x Rosa phoenicia*), cultivated in many countries such as Bulgaria, Turkey, Morocco, Iran, and India. It is a perennial shrub that can reach 50 years of life and reaches its highest productivity around 25 years. Also known as Damask rose, it is grown mainly for the production of essential oil and rose water; however, several commercial products are manufactured from rose flowers [1]:

- Rose essential oil (from fresh and dried flowers) can be utilized as a cosmetic ingredient, flavoring agent for foods, and relaxant agent for aromatherapy [2].
- Rosewater, obtained by hydrodistillation of petals, contains a small percentage of rose oil, and it is employed as a cosmetic ingredient and flavoring agent for foods [3].
- Rose concrete, derived from the extraction of rose petals with organic solvents (e.g., hexane and petroleum ether), is a waxy semi-solid material mainly used to obtain the rose absolute [3,4], while rose absolute is produced by extraction of the concrete with ethanol. It is a red-orange liquid with a rose aroma [4,5].
- Dried flowers: the dried buds and petals of the rose are sold in groceries, herbalist shops, and pharmacies. They are accumulated when there is no profitable production of fresh flowers to be used as an alternative in hydrodistillation [6]. They are used for the preparation of teas and herbal teas, yogurt with antioxidants, and for their relaxing properties.
- Rosehips (dried hips) are employed in traditional medicine for the content of tannins, anthocyanin, phenolic compounds, fatty oil, and organic acids (e.g., ascorbic acid) [7].

From a cosmetic point of view, *Rosa x damascena*-derived ingredients are added in several formulations for their function as skin conditioning agents, antioxidants, cosmetic astringents, and fragrances. Its flower oil, in particular, is used in cosmetic preparations not only as a fragrance but also for its peculiar properties, such as soothing and rebalancing [3].

Regarding flower oil, rose petals contain very little amount of essential oil in comparison with other essential oil plants. For this reason, rose oil is one of the most expensive fragrances sold in the world cosmetic markets, partly due to the lack of natural and synthetic substitutes [8]. Rose oil is generally obtained by hydrodistillation or steam distillation, and it is a very complicated mixture. It is mainly characterized by having a high percentage of monoterpene alcohols, including citronellol, geraniol, nerol, and linalool. With regard to 2-phenyl ethanol, it would be a major oil component, but because of its solubility in water, it is usually partially lost in the distillation waters unless collected as rose water. Many other components in rose oil are present only in trace amounts but are also very important for the overall quality. These components contribute mainly to the perfumery value of rose oil [8]. Literature reports many types of research and analyses carried out on the essential oil and rose water of *Rosa x damascena* [9,10]. Moreover, recently the Cosmetic Ingredient Review (CIR) has published the Safety Assessment of *Rosa x damascena*-derived Ingredients as Used in Cosmetics, evaluating the cosmetic safety of the rose oil and highlighting the presence of some of the 26 fragrance potential allergens (as defined by the Cosmetics Regulation 1223/2009) [11]. In this context, it is well known that the composition of the flower extract varies depending on the extraction method, *Rosa x damascena* type (e.g., Kazanlik), period of flowering, part of the flower used, and collection period. The use of fresh or dried raw material also affects its chemical compositions. Furthermore, the extraction method employed and the storage conditions influence the composition and the qualities of the extract, also in terms of hedonic tone [12,13,14,15,16,17,18]. With regard to this item, it is interesting to note that, by conventional procedures, the obtained plant-derived essential oils can present complex artifact products developed during the water-distillation process due to the influence of high temperature, extraction time, overheating, pH change, etc., inside the distillation stills [19].

In the framework of modern extraction methodologies, Microwave (MW)-Assisted Extractions are considered innovative green techniques, and they have an important role in promoting sustainable processes. Microwave irradiation is a non-contact alternative energetic source (dielectric heating), which can not only make heating more effective and selective but also helps to accelerate energy transfer, start-up, and response to heating control and to reduce thermal gradient, equipment size, and operation units [16]. With the help of microwave-assisted extractive procedures, in particular Solvent-Free Microwave Extraction (SFME) and Microwave Hydrodiffusion and Gravity (MHG), extractions can be completed in minutes instead of hours, with various advantages such as high reproducibility, low solvent, and energy consumption, more compact procedures. Moreover, a higher quality of the final product can be achieved due to a shorter exposure of the matrix to high temperatures of extraction and consequent reduction of thermal degradation compounds [20].

Applying these mild pathways, several compounds of cosmetic interest (e.g., essential oils, antioxidants, pigments, aromas, and other organic compounds) have been efficiently extracted from botanical matrices [21,22]. SFME consists of the MW-assisted dry distillation of a fresh plant matrix without adding water or any organic solvent. The selective heating of the in situ plant water causes tissues to swell and makes the glands and oleiferous receptacles burst. This process thus frees essential oil, which is evaporated by azeotropic distillation with the water naturally present in the plant material [23,24]. The excess water can be refluxed to the extraction vessel to restore and reuse the original water for the plant material. The solvent-free method allows substantial savings of costs in terms of time, energy, and botanical raw material [25].

MHG is an original ‘upside-down’ MW alembic combining MW heating and earth gravity at atmospheric pressure. It exploits the warming of the water contained in the matrix, causing the expansion and consequent rupture of the matrix cells. This phenomenon, known as hydrodiffusion, allows the extract to diffuse outside the matrix. Moreover, MWs increase tissue softness, cell permeability, and cell disruption, thus enhancing the mass transfer within and outside the plant tissue. The extract is collected by gravity, dropping out of the MW reactor via a hole positioned at the bottom of the applicator [26]. It allows for recovery separately, essential oil and a sort of native water enriched with hydrosoluble organic compounds.

Taking into account all these statements, the aim of the research was the evaluation of innovative and green extractive procedures advantageous in a cosmetic context for obtaining high-quality essential oils of *Rosa x damascena* Mill. to be used as fragrances or active ingredients. In the study, as a preliminary analytical screening approach, two microwave-mediated techniques, Solvent-Free Microwave Extraction (SFME) and Microwave Hydrodiffusion and Gravity (MHG), and two conventional procedures, Hydrodistillation (HD) and Steam Distillation (SD), were applied and compared for the extraction of volatile compounds from *rose* fresh petals (cultivated in Italy) to highlight differences and advantages of the applied methods in terms of sustainability, time and energy-saving and yield amount. Furthermore, the quality of the extracts and their suitability for cosmetic applications were evaluated. The chemical composition of the extracts was investigated by GC-MS and GC-FID, and the results obtained were processed by chemometric models employed to analyze the essential oil profile. To point out the correlation between the process and the composition of the extracts, principal component analysis (PCA) related to the chromatographic data was used, allowing to distinguish clustering patterns and identify a classification of the variabilities [27]. 

## 2. Results

**Figure 1:**
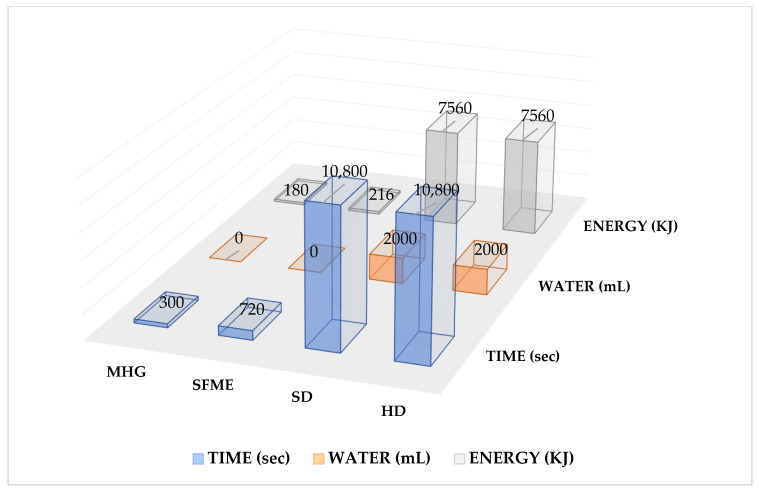
Comparison of the operative conditions of the four methods. MHG—Microwave Hydrodiffusion and Gravity; SFME—Solvent-Free Microwave Extraction; SD—steam distillation; HD—hydrodistillation.

Extracts from petals of *R. x damascena* were obtained using MHG, SFME, SD, and HD affording fragrant yellow, pale oils with 0.28% ± 1.3%, 0.40% ± 1.8%, 0.17% ± 2.7%, and 0.22% ± 3.4% (*w*/*w*) yields, on dry vegetable materials, respectively. Both MW extractions offer shorter extraction times (SFME/HD/SD = 12 min vs. 3 h and MHG/HD/SD = 5 min vs. 3 h), highlighting a great time and energy saving when compared with conventional distillation processes allowing a general better recovery in terms of essential oil yield and number of compounds. The comparison of the data shows the higher efficiency and greenness of the microwave processes (Figure 1).

The GC/FID and GC/MS analysis of the essential oils obtained by the different pathways revealed the presence of 61 constituents (MHG n. 27, SFME n. 45, SD n. 27, and HD n. 25). Compounds are reported in Table 1 as percentages of the total essential oil composition and listed in order of their elution on the Elite-5MS column, and in Table 2, the cumulative percentages related to the different classes of compounds are reported for each extractive method.

*Microwave Hydrodiffusion and Gravity*: major constituents were found to be primary alcohols (61.29%), with a high percentage of 2-phenyl ethanol (59.61%), the most abundant compound. Oxygenated terpenes (monoterpenes and sesquiterpenes) are present in the percentage of 35.5%, where geraniol (16.70%) and citronellol (14.41%) are the most represented, followed by monoterpenic aldehydes in the percentage of 1.55%, being neral (0.67%) and geranial (0.40%) the most represented. Saturated hydrocarbons, accounting for 2.38% of the essential oil, are dominated by nonadecane (1.67%). 

*Solvent-Free Microwave Extraction*: major constituents were found to be primary alcohols (46.89%), from which 2-phenyl ethanol (45.50%) is the most abundant compound. Oxygenated terpenes (monoterpenes and sesquiterpenes) are present in the percentage of 23.4%, whereas caryophyllene oxide (11.66%), geraniol (2.54%), citronellol (2.53%) and α-bisabolol (1.96%) are the most represented. Non-oxygenated terpenes (monoterpenes, sesquiterpenes, and diterpenes) are present in high amount (21.9%) with caryophyllene (10.15%), followed by *trans*-α-bergamotene (2.05%), *trans*-β-farnesene (1.89%), β-selinene (1.53%), and α-selinene (1.20%) as the major constituents. Saturated hydrocarbons account for 3.52% of the extract and are mainly represented by nonadecane (2.23%) and heneicosane (1.04%).

*Steam distillation*: major constituents were found to be oxygenated terpenes (monterpenes and sesquiterpenes) (61.35%), from which citronellol (40.58%) is the most abundant, followed by geraniol (8.48%), linalool (4.62%), isomenthone (1.49%), α-terpineol (1.23%), α-eudesmol (1.14%), and β-eudesmol (1.80%). Primary alcohols account for 25.72% of the extract, mainly represented by 2-phenyl ethanol (25.58%) with a very low amount of benzyl alcohol (0.14%). Saturated hydrocarbons are present in the percentage of 11.17%, nonadecane (5.26%), and heneicosane (4.94%) the most represented. Terpenic aldehydes are present in the percentage of 1.49 %, with neral (0.87%) and geranial (0.62%) as the most abundant compounds. 

*Hydrodistillation:* major constituents were found to be oxygenated terpenes (monterpenes and sesquiterpenes) (51.38%), from which citronellol (30.18%), geraniol (13.36%), β-eudesmol (1.42%), linalol (1.32%), α-terpineol (1.31%), and α-eudesmol (1.16%) are the most abundant compounds. Primary alcohols (27.00%) are dominated by 2-phenyl ethanol (26.90%). Saturated hydrocarbons are present in the percentage of 18.99%, with heneicosane (7.69%), nonadecane (6.42%), and octane (4.45%) as the most represented. Terpenic aldehydes are present in the percentage of 1.03%, with neral (0.56%) and geranial (0.47%).

**Figure 2:**
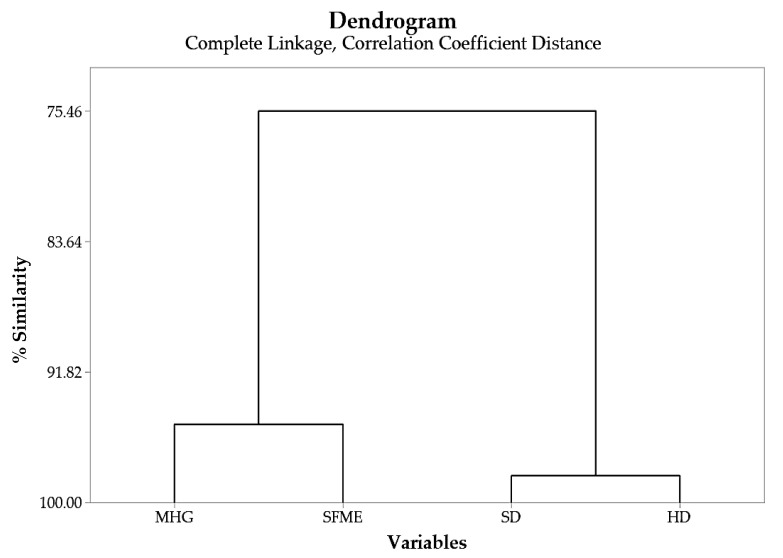
Dendrogram of essential oils composition.

For instance, cluster analysis for the extract composition was conducted due to the numerous compounds which were distillate, and some compounds were correlated to more than one technique. The demonstration confirms the fingerprint of any techniques applied to distillate the composition. Figure 2 shows that the two techniques, SD and HD, are highly correlated in their composition by 98% of the level of similarity, while the other two techniques, MHG and SFME, are correlated in their composition by 95% of the level of similarity. On the other hand, the overall composition of all distillation techniques applied, i.e., MHG, SFME, SD, and HD, are all about 75% similar in their composition.

Furthermore, PCA (Principal Component Analysis) was applied as a potent tool for the reduction of the dimensionality of the correlated variabilities in these numerous compounds in regard to each technique, as this approach has been used in several studies regarding extract profiling. It is a multivariate statistical procedure to reduce the dimensionality of a data set consisting of a large number of interrelated variables [30]. 

The obtained PCA biplot, data not shown, further confirmed the results presented in the dendrogram of Figure 2, where SD and HD are demonstrated to have a similar composition distribution as MHG and SFME techniques.

## 3. Discussion

Steam distillation (SD) and hydrodistillation (HD) represent the most employed techniques for essential oils extraction; however, with some drawbacks such as low extraction yield, loss of some volatile compounds, and being time and energy-consuming [20,31]. Figure 1 resumes a general comparison of the operative conditions of the four methods, as regards time, energy, and water consumption putting into evidence the greater sustainability of these methods. Moreover, MHG and SFME allow for a general higher efficiency (higher yields) and better extract quality, in particular for cosmetic applications. The speediness of MW processes prevents the partial loss of some constituents; this is highlighted by the different compositions of the volatile fraction (Table 1), in particular in regards to benzyl alcohol (10 times higher in MW extracts) and 2-phenyl ethanol (SFME and MHG extract containing double the percentage respect to conventional ones). It can be assumed that the short exposure of the matrix to water (as a solvent) allows for a better repartition of the alcohols in favor of the oily phase, despite their hydrosolubility [32]. This fact seems to be confirmed by the general dramatically higher percentage of the alcoholic fraction obtained by MW extractions vs. conventional ones.

Moreover, as regards oil quality, it is important to highlight that the extracts obtained by MW extraction, especially by SFME, contain an interesting lower level of some potential fragrance allergenic compounds (citronellol, linalool geraniol, neral, and eugenol), as defined by the international organization IFRA (International Fragrance Association) and by the Council of Europe [33,34]. From an applicative point of view and taking into account the antimicrobial activity of both benzyl alcohol and 2-phenyl ethanol [35,36], these results seem to indicate a potential use of the MW extracts to enhance the preservative action in personal care products. The mildness of the SFME procedure can be inferred from the data obtained by GC-FID and GC-MS analysis that show the presence of many aromatic compounds in the extracts obtained with MW methods, which are not obtained with conventional ones. In particular, the composition of the SFME extract, with the presence of 45 compounds compared to the other techniques, probably confers to this product a different hedonic tone, potentially useful in cosmetic fragrances. 

## 4. Materials and Methods

### 4.1. Chemicals

Octyl octanoate (98%), alkane mix (C6–C35), and anhydrous sodium sulfate were obtained by Sigma-Aldrich, Inc. (St. Louis, MO, USA). Diethyl ether was purchased from Merck (Darmstadt, Germany). Ultrapure water (LC-MS grade) was produced using the Milli Q-Milli RO system, Millipore (Burlington, MA, USA). 

### 4.2. Plant Materials

Fresh petals of *Rosa x damascena* Mill. (Kazanlik type) were kindly provided by JB Rose Farm, Ronco Scrivia, (Genova, Italy, 29.54 N ′18.6 E). Samples were collected at the flowering stage and immediately refrigerated at +4 °C and subsequently stored at −20 °C until extraction. The initial moisture content (relative humidity) of fresh flowers was determined to be 78.62% ± 2.2% by a Sartorius moisture analyzer (Sartorius AG, Goettingen, Germany). All measurements were made in triplicate, and the average results were reported. Triplicate samples of fresh petals of *Rosa x damascena* Mill. (30–50 g, depending on the technique used), to which octyl octanoate (30 mg) was added as internal standard [23], were subjected to the following extractive procedures.

### 4.3. Extractive Procedures

#### 4.3.1. MHG Apparatus and Procedure

Triplicate samples of fresh rose petals (30 g), without the addition of any solvents or water, were irradiated at different times and temperatures to optimize the extractive conditions. The best results were obtained with an extraction time of 5 min (1 min up to 100 °C and held for 4 min). The apparatus used for this green technique is a multimode microwave prototype with a maximum delivered power of 600 Watts. It consists of a cavity equipped with a specially designed Pyrex reactor, a magnetron operating at 2.45 GHz, two optical fibers for temperature measurement, and a feedback control unit that allows to manage and modulate different parameters of the process such as emitted power, time and temperature. Moreover, the furnace presents two holes on the walls of the cavity, which allow exploiting the prototype applicator for several applications (also for continuous flow reactions and chemical synthesis). Accordingly, with the method proposed by Chemat and coworkers [37], essential oil forms a film on the surface of the water collected continuously in a receiving flask (similar to a separatory funnel). The essential oil was collected, dried under anhydrous sodium sulfate, and stored in dark sealed vials at −20 °C until analyses.

#### 4.3.2. SFME Apparatus and Procedure

Triplicate samples of fresh rose petals (30 g), without the addition of any solvents or water, were irradiated at different times and temperatures to optimize the extractive conditions. The best results were obtained with an extraction time of 12 min (2 min up to 100 C and held at this temperature for 10 min). The solvent-free extraction was performed using a single-mode microwave scientific reactor (Discover^®^, CEM Corporation, Matthews, NC, USA) with a maximum power of 300 Watts delivered in 10 W increments. During experiments, time, temperature, and power were controlled by specific software. The apparatus was equipped with a cooling system using compressed air. The temperature was monitored by an optical fiber directly inserted into the sample container and regulated by a power feedback control from 0 to 300 Watts. The sample vessel was equipped with a modified Clevenger apparatus, according to Chemat et al. [31]. Essential oil and aromatic water (hydrosol) were simply separated by decantation. The essential oil was collected, dried under anhydrous sodium sulfate, and stored in dark sealed vials at −20 °C until analyses.

#### 4.3.3. SD Apparatus and Procedure

Triplicate samples of fresh rose petals (50 g) were steam distilled for 3 h with 2 L of distilled water (Heating isomantel, 700 Watts). Temperature (100 °C) was measured by a digital thermocouple thermometer (Comark, city, UK). According to the Italian Pharmacopoeia, plant material is placed on the perforated grid, then water steam is released from the steam boiler to the distillation vessel and passes through the plant material [38]. The essential oil is separated from vegetable material by the diffusion process and comes out with steam from the water-cooled condenser to the Florentine flask, thereby producing the hydrosol [39]. The 3 h of distillation time started when the first drop of liquid condensed in the cooling apparatus was dropped into the Florentine flask. The essential oil was collected, dried under anhydrous sodium sulfate, and stored in dark sealed vials at −20 °C until analyses. 

#### 4.3.4. HD Apparatus and Procedure 

Rose petals (50 g) were submitted to hydrodistillation using a Clevenger-type apparatus according to the Italian Pharmacopoeia [38] and extracted for 3 h with 2 L of distilled water (Heating isomantel, 700 Watts). Temperature (100 °C) was measured by a digital thermocouple thermometer (Comark City, UK). The 3-h distillation time started when the first drop of liquid was condensed in the cooling apparatus and dropped into the graduated burette. The essential oil was collected and dried under anhydrous sodium sulfate and stored in dark sealed vials at −20 °C until analyses. 

### 4.4. GC-FID and GC-MS Analysis

The analyses of extracts were performed according to Robustelli della Cuna et al. [40]. 

#### Identification of the Components of the Extracts

The identification of the volatile oil compounds was performed using retention indices (RI) and mass spectra, according to Adams [28], and by comparison with a NIST database mass spectral library [29]. The relative amount of each component was expressed as percent peak area relative to total peak area from GC/FID analyses of the whole extracts. The quantitative data were obtained from GC/FID analyses by internal standard method and considering an equal response factor for all compounds.

### 4.5. Chemometrics and Statistical Analysis

Statistical analysis for the composition of the volatile fraction was performed using Minitab19^®^ software. Data were scaled and interpreted in percentage before processing, i.e., each column of the data matrix was the mean of triplicate analysis and normalized to the standard deviation.

## 5. Conclusions

The advantages of microwave extractive procedures, in particular of SFME, can be deduced from the results obtained by the comparison of the four extraction methods selected. Extraction times are significantly reduced; rose oil recoveries are higher, and energy consumption is meaningfully lower. From the data obtained by GC-FID and GC-MS analysis, it was possible to ascertain the presence of many aromatic compounds in the extracts obtained with microwave techniques, which are not obtained with conventional methods. These data allowed to put into evidence a greater cosmetic interest and safety of the obtained MW fragrances in terms of a reduced number of potential allergens and a significantly higher amount of phenyl ethanol, a useful compound with antimicrobial properties.

## Figures and Tables

**Table 1:** Percent composition of *Rosa x damascena* fresh petals essential oils.

**#**	**Compound ^a^**	**RI ^b^**	**RI ^c^**	MHG	SFME	SD	HD	Identification ^e^
				**% ^d^**	**%**	**%**	**%**	
1	Octane	800	800	-	-	0.40 ± 0.8	4.45 ± 0.6	STD, RI
2	2-Hexenal	854	852	-	-	0.28 ± 0.4	0.25 ± 0.8	RI, MS
3	Heptanal	902	902	-	-	0.56 ± 0.9	0.24 ± 0.7	RI, MS
4	α-pinene	939	931	-	-	0.28 ± 0.7	0.51 ± 0.4	RI, MS
5	Benzaldehyde	960	958	0.26 ± 0.2	0.02 ± 0.4	0.50 + 0.7	0.61 ± 0.2	RI, MS
6	β-myrcene	991	991	0.03 ± 0.2	0.51 + 0.2	0.24 + 0.5	0.30 ± 0.1	RI, MS
7	Benzyl alcohol	1032	1033	1.68 ± 0.4	1.39 + 0.2	0.14 + 0.6	0.10 ± 0.5	RI, MS
8	Phenylacetaldehyde	1042	1042	0.23 ± 0.3	0.12 ± 0.5	0.47 ± 0.8	0.46 ± 0.7	RI, MS
9	*trans*-sabinene hydrate	1070	1066	0.28 ± 0.5	-	-	-	RI, MS
10	Linalool	1094	1099	0.45 ± 0.4	-	4.62 ± 0.4	1.32 ± 0.2	RI, MS
11	2-phenyl ethanol	1139	1139	59.61 ± 0.5	45.50 ± 0.6	25.58 ± 0.7	26.90 ± 0.9	RI, MS
12	Isomenthone	1162	1169	0.26 ± 0.4	0.19 ± 0.3	1.49 ± 0.3	0.93 ± 0.3	RI, MS
13	Unidentified	-	1201	0.14 ± 0.1	-	-	-	-
14	α-terpineol	1189	1215	0.25 ± 0.2	0.12 ± 0.5	1.23 ± 0.5	1.31 ± 0.3	RI, MS
15	Verbenone	1204	1208	0.19 ± 0.2	-	-	-	-
16	Citronellol	1230	1229	14.41 ± 0.3	2.53 ± 0.3	40.58 ± 0.9	30.18 ± 0.4	RI, MS
17	Geranial	1249	1241	0.40 ± 0.2	-	0.62 ± 0.4	0.47 ± 0.9	RI, MS
18	Geraniol	1253	1256	16.70 ± 0.4	2.54 ± 0.4	8.48 ± 0.5	13.36 ± 0.5	RI, MS
19	Neral	1270	1271	0.67 ± 0.5	0.11 ± 0.3	0.87 ± 0.7	0.56 ± 0.1	RI, MS
20	Eugenol	1359	1355	0.38 ± 0.4	-	0.12 ± 0.4	0.28 ± 0.6	RI, MS
21	Ethyl nerolate	1359	1360	-	0.64 ± 0.2	-	-	RI, MS
22	Hexyl hexanoate	1387	1387	-	0.25 ± 0.3	-	-	RI, MS
23	Isocariophyllene	1435	1408	-	0.26 ± 0.3	-	-	RI, MS
24	*cis*-α-bergamotene	1411	1417	-	0.23 ± 0.2	-	-	RI, MS
25	*trans*-caryophyllene	1419	1421	0.11 ± 0.2	10.15 ± 0.4	-	-	RI, MS
26	*trans*-α-bergamotene	1432	1437	-	2.05 ± 0.6	-	-	RI, MS
27	*trans*-β-farnesene	1457	1458	-	1.89 ± 0.3	-	-	RI, MS
28	aromadendrene	1463	1463	-	0.81 ± 0.2	-	-	RI, MS
29	γ-cadinene	1477	1478	-	0.17 ± 0.3	-	-	RI, MS
30	Germacrene D	1482	1482	0.06 ± 0.4	-	0.03 ± 0.8	-	RI, MS
31	α-curcumene	1483	1484	-	0.40 ± 0.1	-	-	RI, MS
32	β-selinene	1490	1488	-	1.53 ± 0.2	-	-	RI, MS
33	α-selinene	1498	1496	-	1.20 ± 0.3	-	-	RI, MS
34	β-bisabolene	1506	1510	-	0.53 ± 0.2	-	-	RI, MS
35	*trans-*β-guaiene	1503	1522	-	0.15 ± 0.2	-	-	RI, MS
36	δ-cadinene	1523	1525	-	0.22 ± 0.3	-	-	RI, MS
37	α-cadinene	1539	1537	-	0.62 ± 0.4	-	-	RI, MS
38	Unidentified	-	1540	-	0.35 ± 0.2	-	-	RI, MS
39	Selina-3,7(11)-diene	1547	1544		0.88 ± 0.3	-	-	RI, MS
40	Elemol	1548	1551	0.24 ± 0.3	-	-	-	RI, MS
41	Germacrene B	1561	1559	-	0.19 ± 0.5	-	-	RI, MS
42	Nerolidol	1563	1565	-	0.53 ± 0.2	-	-	RI, MS
43	Cariophyllene oxide	1583	1587	-	11.66 ± 0.7	-	-	RI, MS
44	Ledol	1590	1606	-	0.23 ± 0.8	-	-	RI, MS
45	Unidentified	-	1612	-	3.26 ± 0.4	-	-	-
46	10-epi-γ-eudesmol	1619	1621	-	0.79 ± 0.2	-	-	RI, MS
47	γ-eudesmol	1630	1635	0.12 ± 0.2	0.38 ± 0.5	0.40 ± 0.7	0.39 ± 0.2	RI, MS
48	Caryophylladienol II	1641	1639	-	0.54 ± 0.3	-	-	RI, MS
49	β-eudesmol	1651	1653	0.30 ± 0.2	-	1.8 ± 0.6	1.42 ± 0.5	RI, MS
50	α-eudesmol	1664	1656	0.41 ± 0.2	0.34 ± 0.2	1.14 ± 0.5	1. 16± 0.5	RI, MS
51	β-bisabolol	1675	1675	-	0.71 ± 0.5	-	-	RI, MS
52	α-bisabolol	1680	1685	-	1.96 ± 0.3	-	-	RI, MS
53	Eudesm-7(11)-en-4-ol	1700	1699	-	0.13 ± 0.3	-	-	RI, MS
54	Cryptomeridiol	1814	1815	0.44 ± 0.6	-	-	-	RI, MS
55	Phytone	1845	1846	-	0.28 ± 0.1	-	-	RI, MS
56	Nonadecane	1900	1900	1.67 ± 0.2	2.23 ± 0.5	5.26 ± 0.6	6.42 ± 0.6	STD, RI
57	Totarene	1923	1921	-	0.11 ± 0.5	-	-	STD, RI
58	Unidentified	2195	1999	-	-	0.14 ± 0.5	0.27 ± 0.1	-
59	Heneicosane	2100	2099	0.49 ± 0.3	1.04 ± 0.3	4.94 ± 0.6	7.69 ± 0.4	STD, RI
60	Tricosane	2300	2299	0.22 ± 0.2	0.25 ± 0.2	0.43 ± 0.5	0.43 ± 0.2	STD, RI
61	Pentacosane	2500	2500	-	-	0.14 ± 0.2	-	STD, RI

MHG—Microwave Hydrodiffusion and Gravity; SFME—Solvent-Free Microwave Extraction; SD—steam distillation; HD—hydrodistillation. ^a^ Compounds are listed in order of elution from an Elite-5 column. ^b^ Kovats Retention Indices according to Adams [28]. ^c^ Retention index determined on an Elite-5 column using a homologous series of *n*-alkanes. ^d^ mean ± SD of three determinations. ^e^ Method of identification: STD—pure compound; MS—mass spectrum in comparison with library [29]; RI—retention indices in agreement with literature values.

**Table 2:** Cumulative percentages related to the different classes of compounds for each extractive method.

Analytical Compounds				MHG %	SFME %	SD %	HD %
**NOT TERPENIC COMPOUNDS**			Saturated hydrocarbons	2.38	3.52	11.17	18.99
			Primary Alcohols	61.29	46.89	25.72	27.00
			Aldehydes	0.49	0.14	1.81	1.56
			Ketones	-	0.28	-	-
			Esters	-	0.25	-	-
			**Total**	**64.16**	**51.08**	**38.7**	**47.55**
**TERPENIC COMPOUNDS**	MONOTERPENES	Oxygenated	Alcohols	32.47	5.19	55.03	46.45
			Aldehydes	1.07	0.11	1.49	1.03
			Ketones	0.45	0.19	1.49	0.93
			Esters		0.64		
			**Total oxygenated mono**	**33.99**	**6.13**	**58.01**	**48.41**
		Non-oxygenated		0.03	0.51	0.52	0.81
		**Total mono**		**34.02**	**6.64**	**58.53**	**49.22**
	SESQUITERPENES	Oxygenated	Alcohols	1.51	5.61	3.34	2.97
			Epoxydes		11.66		
			**Total oxygenated sesqui**	**1.51**	**17.27**	**3.34**	**2.97**
		Non-oxygenated		0.17	21.28	0.03	-
		**Total sesqui**		**1.68**	**38.55**	**3.37**	**2.97**
	DITERPENES	Non-oxygenated		-	0.11	-	-
			**Total oxygenated terpens**	**35.5**	**23.4**	**61.35**	**51.38**
		**Total Terpenes**		**35.7**	**45.3**	**61.9**	**52.19**
**UNIDENTIFIED**				0.14	3.61	0.14	0.27

## Data Availability

The data presented in this study are available on request from the corresponding author. The data are not publicly available due to privacy.

## Sample Availability

Samples of the compounds are available from the authors.
